# Flow-volume loops derived from three-dimensional echocardiography: a novel approach to the assessment of left ventricular hemodynamics

**DOI:** 10.1186/1476-7120-6-13

**Published:** 2008-04-04

**Authors:** Kambiz Shahgaldi, Emil Söderqvist, Petri Gudmundsson, Reidar Winter, Jacek Nowak, Lars-Åke Brodin

**Affiliations:** 1 Department of Cardiology, Karolinska University Hospital Huddinge, Stockholm, Sweden; 2 St. Jude Medical, Järfälla, Sweden; 3 Health Care Group, Malmö, Sweden; 4 Department of Clinical Physiology, Karolinska University Hospital Huddinge, Stockholm, Sweden; 5 School of Technology and Health, Royal Institute of Technology, Flemingsberg, Stockholm, Sweden

## Abstract

**Background:**

This study explores the feasibility of non-invasive evaluation of left ventricular (LV) flow-volume dynamics using 3-dimensional (3D) echocardiography, and the capacity of such an approach to identify altered LV hemodynamic states caused by valvular abnormalities.

**Methods:**

Thirty-one patients with moderate-severe aortic (AS) and mitral (MS) stenoses (21 and 10 patients, respectively) and 10 healthy volunteers underwent 3D echocardiography with full volume acquisition using Philips Sonos 7500 equipment. The digital 3D data were post- processed using TomTec software. LV flow-volume loops were subsequently constructed for each subject by plotting instantaneous LV volume data sampled throughout the cardiac cycle vs. their first derivative representing LV flow. After correction for body surface area, an average flow-volume loop was calculated for each subject group.

**Results:**

Flow-volume loops were obtainable in all subjects, except 3 patients with AS. The flow-volume diagrams displayed clear differences in the form and position of the loops between normal individuals and the respective patient groups. In patients with AS, an "obstructive" pattern was observed, with lower flow values during early systole and larger end-systolic volume. On the other hand, patients with MS displayed a "restrictive" flow-volume pattern, with reduced diastolic filling and smaller end-diastolic volume.

**Conclusion:**

Non-invasive evaluation of LV flow-volume dynamics using 3D-echocardiographic data is technically possible and the approach has a capacity to identify certain specific types of alteration of LV flow-volume pattern caused by valvular abnormalities, thus reflecting underlying hemodynamic states specific for these abnormalities.

## Background

Measurement of left ventricular end-systolic (ES) as well as end-diastolic (ED) volumes and LV ejection fraction (EF) is a well-established procedure in the evaluation of cardiac function. Two-dimensional (2D) echocardiography is the most commonly used imaging modality in the diagnosis of heart diseases and the primary tool for the assessment of LV function and volumes. However, 2D echocardiography has limited reproducibility due to the fact that algorithms for the calculation of LV volumes and EF are based on certain geometrical assumptions that do no necessarily hold true in different clinical scenarios, as for example, in the presence of regional wall motion or structural abnormalities and dilated ventricles with changed geometry [1-3]. Nevertheless, 2D-echocardiography-based assessment of EF and LV volumes still not only provides important diagnostic information, but can also be used as a therapeutic guidance and prognostic instrument [4,5].

Three-dimensional echocardiography offers the possibility of a more accurate assessment of LV volumes and EF. Studies performed hitherto have consistently demonstrated improved accuracy of LV volumetry by 3D echocardiography [6-13] compared to 2D technique [7,14,15]. One of the disadvantages of the 3D-echocardiographic approach is the fact that it is rather time consuming due to the necessity of manual tracing of endocardial borders in several LV slices [11]. However, the newly introduced real-time 3D technique with semi-automated border tracing allows faster acquisition of 3D data sets with good image quality and faster data analysis. Nevertheless, the assessment of LV function from end-systolic and end-diastolic LV volumes remains in its essence rather crude and does not provide any detailed hemodynamic data from the LV filling and ejection phases.

The most reliable method for the evaluation of LV contractility is the simultaneous, real-time measurement of LV pressure and volume and the analysis of thus created pressure-volume loop diagram [16]. However, the procedure has a disadvantage of being invasive and is therefore seldom used in clinical practice. An alternative, non-invasive way to the assessment of LV contractility has been proposed and involves analysis of the relationship between sphygmomanometrically measured systolic blood pressure (substitute of LV end-systolic pressure) and echocardiographically derived LV end-systolic volume at rest and during exercise [17]. Recently, Söderqvist et al. described a new non-invasive approach to the assessment of LV function based on the analysis of LV flow-volume relationship in time domain using estimates of LV volume and flow derived from echocardiograpic variables [18]. The concept of cardiac flow-volume measurements originates from the flow-volume analysis that has been for long generally applied as a clinical measure of lung function and differentiation between obstructive and restrictive disorder. Similarly, the LV flow-volume relationship throughout the cardiac cycle presented as a flow-volume loop can be expected to show different characteristic pattern in different specific hemodynamic situations and to provide thereby useful diagnostic information and therapeutic guidance.

Three-dimensional echocardiography provides possibility of a direct measurement of LV volumes and the first derivative of LV volume would describe blood flow in and out of the LV. Hence, by using volume and flow data extracted from the 3-D data set acquired throughout the cardiac cycle, left ventricular flow-volume loops can be constructed. The aim of the present study was to explore the feasibility of non-invasive evaluation of LV flow-volume dynamics using 3D echocardiography, and to assess the diagnostic potential of such flow-volume loops in two different types of hemodynamic pathologies, i.e. the obstructive disorder due to aortic stenosis and the restrictive state resulting from mitral stenosis.

## Methods

The study population consisted of 41 subjects. Thirty-one of them (17 women and 14 men, aged 72 ± 12 years) were patients referred to the Department of Cardiology, Malmö University Hospital for echocardiographic evaluation of mitral or aortic stenosis, and 10 subjects (3 women and 7 men, aged 32 ± 3 years) were healthy volunteers. In addition to standard 2D echocardiography, 3D echocardiography was performed in all subjects. The severity of the mitral and aortic stenosis was defined according to the clinical practise guidelines at Malmö University Hospital and in agreement with ACC/AHA guidelines [19] as mild, moderate, moderate-severe, or severe. The assessment of the severity of the valvular stenoses was performed using a combination of qualitative and quantitative analysis based on echocardiographic spectral and colour Doppler measurements. The study was approved by the Ethics Committee of the Lund University Hospital and all study subjects gave their informed consent to participate.

### 3D echocardiography

**Figure 1:**
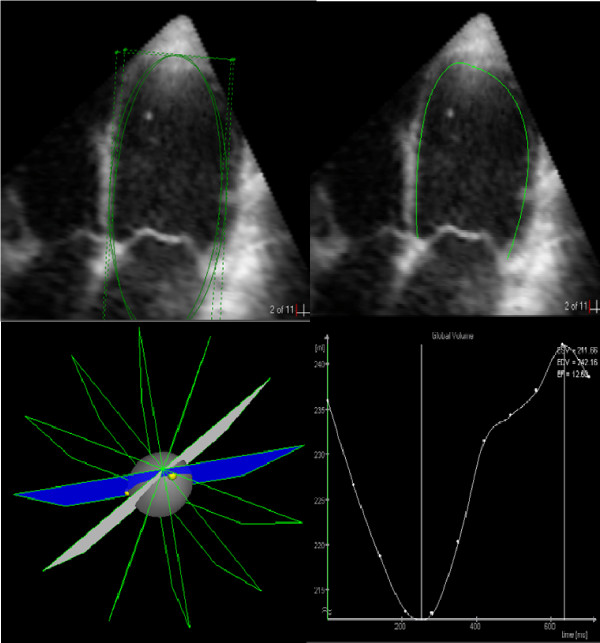
**Analysis of LV volumes using 3D-echocardiography.** In the selected LV image (upper left), the algorithm (4D LV-Analysis CRT 1.0, TomTec) provides semi-automated delineation of the endocardial border (upper right). By superposition of LV contours from 6 insonation planes (lower left) in 3D space, the LV volumes are calculated and the graphic presentation of LV volume changes in time can be obtained (lower right).

All 3-D echocardiographic examinations were performed by the same experienced sonographer with the patient in the left lateral position. 3D images were acquired over 4 consecutive cardiac cycles during a breath hold from the apical window using Sonos 7500 equipment and a full matrix array ultrasonographic (x4) transducer (Philips, Andover, Mass., U.S.A.). The sampling frequency of 3D data was 15 – 24 frames/s. The acquired data sets were stored on CD-ROM disks and transferred to a standard PC for subsequent off-line analysis using commercially available software 4D LV-Analysis CRT 1.0 (TomTec, Unterschlessheim, Germany). The analysis started with the definition of long axis plane in apical 5-chamber view, whereupon 6 long axis image planes of LV were automatically generated. In each of the 6 image planes, the position of mitral valve, aortic valve and apex was marked at the end of systole (smallest LV cavity size) and diastole (largest LV cavity size). The semi-automated border detection algorithm provided subsequently a delineation of LV endocardial border with a preconfigured ellipse that was manually adjusted and the total endocardial border in all frames could be defined. The analysis program allowed then a reconstruction of LV volumes as a dynamic surface-rendered image in which LV wall motion was shown three-dimensionally. A graph describing LV volume changes throughout the cardiac cycle was also obtained (Fig. 1). Left ventricular end-diastolic (LVEDV) and end-systolic (LVESV) volumes as well as LV ejection fraction (LVEF) were automatically provided by the software from the acquired 3D data.

### Construction of LV flow-volumes loops

Using 3D-data set, LV volumes were determined for each frame throughout the cardiac cycle and their respective first derivatives were calculated using Matlab software (version 6.5, TheMathWorks Inc. Natick, MA, U.S.A.). All volume variables were then adjusted for body surface area and plotted against its first derivative (describing LV flow) for each cardiac cycle to create LV flow-volume loops. Subsequently, an average flow-volume loop for each examined patient group was constructed. The created flow-volumes loops are limited to one cardiac cycle and lack time scale having LV volume as *x*-axis. Consequently, expressions pointing to diastolic/systolic early/late flows mean flows at the respective small/large left ventricular volumes.

### Statistics

All values are presented as mean ± SD. The Student's *t*-test for unpaired samples was used as appropriate.

## Results

3D-echocardiographic data were acquired from all 41 subjects. However, in 3 patients with aortic stenosis (AS), the image quality was poor and their 3D data have been excluded from analysis.

The clinical characteristics of the study population are presented in Table 1. Significant AS or mitral valvular stenosis (MS) was found in 74 % of the patients, whereas in the resting 26% of the subjects no cardiac pathology was found. The distribution of the valvular diseases, 3D-echocardiographic volumes, as well as blood pressure and heart rate data in the study population is presented in Table 2. As could be expected, the patients with MS showed smaller LVEDV than the subjects without any valvular pathology (p < 0.05), and also LVEF was smaller both in the patients with AS and MS, as compared to non-affected individuals (p < 0.05). Also, the patients with MS had higher heart rate than those with AS (p < 0.05) and the subjects without any valvular disease (p < 0.001).

**Table 1:** Clinical characteristics of the study subjects.

Sinus rhythm	71%
Dilatead left ventricle	13%
Dilated left atrium	50%
Heart failure	24%
Previous AMI	11%
Previous PCI	3%
Previous CABG	16%
Diabetes	5%
Significant valvular disease	74%
Beta-blocker	37%
ACE inhibitor	26%

AMI: acute myocardial infarction, PCI: percutaneous coronary intervention, CABG: coronary artery bypass grafting, ACE: angiotensin converting enzyme

**Table 2:** Distribution of the 3D-echocardiographic data, blood pressure and heart rate in the study population.

**Valvular disease**	**Number of subjects**	**Severity of valvular disease**			**LVEDV** (ml)	**LVESV** (ml)	**LVEF** (%)	**BP**		**HR** (beats/min)
		**M**(n)	**M-S**(n)	**S**(n)				**systolic** (mm Hg)	**diastolic** (mm Hg)	
AS	18	7	2	9	110 ± 46	63 ± 38	47 ± 17*	154 ± 49	75 ± 12	74 ± 14*
MS	10	7	1	2	83 ± 26*	42 ± 12	48 ± 13*	139 ± 22	78 ± 17	88 ± 13
None	10	0	0	0	110 ± 20	46 ± 10	59 ± 4	124 ± 15	72 ± 9	66 ± 5***

M: moderate, M-S: moderate to severe, S: severe, BP: blood pressure, HR: heart rate, * p < 0.05 vs. LVEDV/LVEF – no valvular disease, and heart rate – MS; *** p < 0.001 vs. Heart – MS

**Figure 2:**
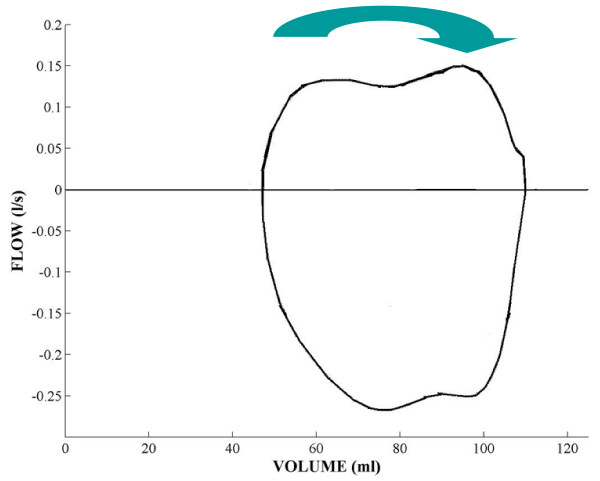
**A schematic illustration of flow-volume loop during one cardiac cycle.** The loop proceeds in clockwise direction. The upper part of the loop (above zero flow) describes flow-volume relationship during diastole, and the lower part of the loop (below zero flow) shows the relationship during systole.

A schematic diagram of a LV flow-volume loop during one cardiac cycle is presented in Fig. 2. The loop proceeds in clockwise direction with decreasing volume during LV ejection and increasing LV volumes during diastolic filling. The positive flow values indicate flow into the left ventricle during diastole and the negative values flow out of the left ventricle during systolic ejection. The curve indicates maximal LV volume at the end of diastole and minimal LV volume at the end of systole, and the diastolic E- and A-wave can also be discerned.

**Figure 3:**
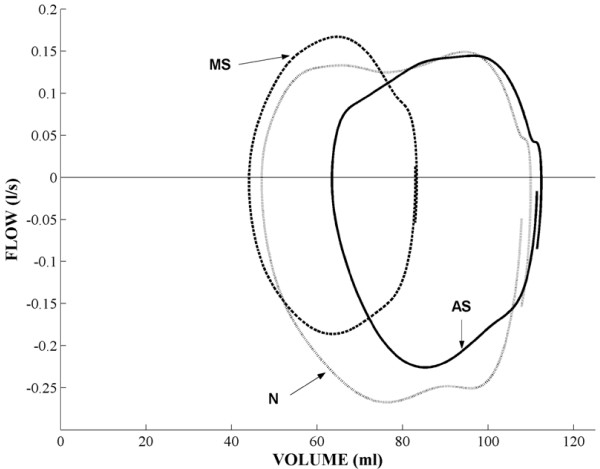
**Average LV flow-volume loops in normal individuals (N), and patients with valvular abnormalities (MS and AS).** Note the characteristic alteration of the loop form and the position of the loop in the flow-volume diagram in the two patient groups, as compared to healthy subjects.

The average LV flow-volume loops in patients with severe valvular abnormalities and in individuals without valvular pathology are presented in Fig. 3. As can be seen from the figure, the flow-volume relationships were altered in both left-sided cardiac valvular abnormalities and differed from the typical loop in subjects with unaffected valves. In addition, flow-volume pattern differed between the two studied left-sided valvular pathologies as well. The patients with AS displayed easily discernible slower systolic volume decrease and lower ejection fraction despite maintained LVED volume, thus reflecting hampered systolic ejection. On the other hand, in the patients with MS, the flow-volume loop indicated smaller LVED volumes and smaller ejection fraction implying reduced diastolic LV filling (Fig. 3 and Table 2).

## Discussion

Analysis of invasively derived LV pressure-volume loops is a very useful and reliable method for monitoring of myocardial contractility (16) and an appreciated research tool whenever detailed information about the dynamics of LV performance is needed. However, the method suffers from its invasiveness that limits its general applicability in clinical cardiology. Therefore, there has been a long-felt need of most cardiologists for an alternative, non-invasive approach to the evaluation of myocardial contractility and it has been proposed that non-invasive measurement of changes in end-systolic pressure/volume ratio (systolic pressure by cuff sphygmanometer/end-diastolic volume by biplane Simpson's rule method) from rest to peak exercise could be used as a sensitive indicator of left ventricular function [20]. Based on this concept, a new non-invasive echocardiographic method has been developed that allows assessment of LV function by analysis of the dynamics of the systolic pressure/end-diastolic volume relationship during physical stress [21], pacing [22], or pharmacological stress with dobutamine [23]. The concept of LV flow-volume loop presented in the present study creates basis for yet another new, non-invasive approach to the assessment of LV function by focussing on a continuous quantitative evaluation of flow-volume relationship throughout the entire cardiac cycle. In addition to quantifiable variables such as flow and volume, other characteristics of the flow-volumes loops such as, for example, the course of ventricular filling and ejection, and the overall form of the flow-volume loop may also be evaluated qualitatively.

The concept of LV flow-volume loop provides a new and different from pressure-volume loop approach to the estimation of the dynamics of cardiac performance. The approach was originally proposed by Söderqvist et al. [18] and was based on the use of estimates of LV flow and volume derived from 2D echocardiography images. Even if only estimates of LV volumes were used in the above-mentioned study, the obtained results clearly proved the technical feasibility of non-invasive monitoring of LV flow-volume relationship. In contrast to study of Söderqvist et al. [18], the LV volumes the present study was measured directly using 3D echocardiography. The inherent limitations of the 2D volume calculations using Simpson's formula based on certain geometrical assumptions was thus eliminated, and consequently, also the present calculation of the first derivative of LV volume representing time-dependent LV flow was more accurate. Indeed, the 3D-echocardiographic quantification of LV volumes has been shown to be highly reproducible and accurate when compared with 2D echocardiography [3,7,15,24,25], magnetic resonance imaging [3,7,12,26-28], or single photon emission tomography [27]. Hence, the flow-volume curves based on the 3D-echocardiographic measurements can be expected to provide reliable information about LV flow events and simultaneous changes in ventricular volume, complementing thereby other hemodynamic information that may be needed in specific clinical or research settings.

The hemodynamic information that can be retrieved from flow-volume loops describes the dynamics of flow-volume events. For this reason, the present approach is superior to the standard methods of functional LV assessment that use only end-diastolic and end-systolic volumes for calculation of LV ejection fraction. Incorporation of the dynamics of LV flow and volume in the same loop provides a possibility of a new and more detailed assessment of cardiac hemodynamics from different point of view. The continuous information about LV flow and volume expressed by flow-volume loop provides the prerequisites for hemodynamic pattern recognition in health and disease. In this process, the qualitative assessment of flow-volume loop form can be used as a tool for a rapid evaluation of cardiac hemodynamics and differentiation between normal state and different cardiac pathologies, as for example, significant valvular diseases that would change the normal pattern of LV filling and emptying. In the course of disease in any given patient, the sequential comparison of the respective loop forms and positions in the diagram can be easily performed and may provide valuable additional information and therapeutic guidance.

The present results demonstrate that flow-volume loops based on 3D echocardiography measurements indeed have a potential to separate groups of different hemodynamic states. The characteristics of the average flow-volume loops obtained in normal subjects and in patients with AS an MS differed and were easily distinguishable. The patients with AS demonstrated a typical "obstructive" pattern with lower flow values during early systole and larger end-systolic volume as compared to normal pattern. On the other hand, in the patients with MS a "restrictive" pattern with reduced diastolic filling was observed. The prevailing hemodynamic state may thus be rapidly determined by recognition of a specific typical pattern of flow-volume relationship.

The present methodological approach has, however, some limitations. The major limitation is caused by the relatively low 3D-echocardiographic sampling rate ranging from15 to 24 Hz. This would increase the variability of the flow-volume loops. As a consequence, although providing a typical pattern for a group of patients with a specific cardiac pathology, the loops may not yet be applicable on the individual basis. The other important limitation lies in the fact that the same data set is used for calculation of both volume and flow, the latter being calculated as the first derivative of volume. This limitation can be overcome by using integrated 3D colour Doppler in the LV outflow tract for flow measurements and 3D grey scale data for continuous measurement of LV volume. Finally, the image quality is also a limiting factor.

## Conclusion

3D echocardiography offers the possibility of non-invasive estimation of the dynamics of LV flow-volume relationship during cardiac cycle. The approach provides new information about LV hemodynamics and function, and has a capacity to differentiate between normal LV hemodynamics and specific types of alterations of flow-volume relationships caused by valvular abnormalities, such as aortic and mitral stenoses. The method has a potential to provide additional hemodynamic information in the evaluation of cardiac and valvular function.

## Competing interests

The author(s) declare that they have no competing interests.

## Authors' contributions

RW and LÅB initiated the study. RW supervised the study and participated in the interpretation of the results and manuscript preparation. KS performed measurements, made all data conversions, plots and calculations from ultrasound data, and participated in the preparation of the manuscript. PG participated in data collection and interpretation of the results. ES participated in data conversion, creation of plots and calculations from ultrasound data. JN performed statistical analysis and was responsible for final manuscript. All authors read and approved the final manuscript.
